# Supplementary material for: Genetic architecture and lifetime dynamics of inbreeding depression in a wild mammal
Source: Nat Commun. 2021 May 20;12:2972. doi: 10.1038/s41467-021-23222-9 (PMC8138023; doi:10.1038/s41467-021-23222-9)
Supplement: Supplementary file 1 — Supplementary Information [file 41467_2021_23222_MOESM1_ESM.pdf]

Supplementary Information

Genetic architecture and lifetime dynamics of inbreeding depression in a wild mammal

Authors names and addresses:

Stoffel, M.A.[1*], Johnston, S.E.[1], Pilkington, J.G.[1], Pemberton, J.M[1]

[1]Institute of Evolutionary Biology, School of Biological Sciences, University of Edinburgh, Edinburgh, EH9 3FL, United Kingdom

* Corresponding author:
Martin A. Stoffel
Postal address: Institute of Evolutionary Biology, University of Edinburgh, Edinburgh, EH9 3FL, UK
E-mail: martin.stoffel@ed.ac.uk

Supplementary Figures

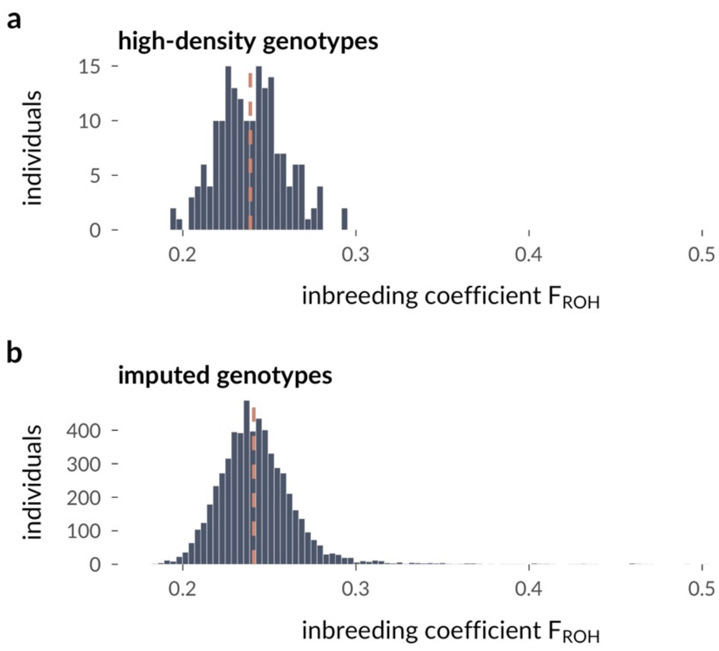

Supplementary Figure 1: Distribution of F_{ROH} for individuals with non-imputed (high-density) and imputed SNPs. a Distribution of inbreeding coefficients F_{ROH} of 181 individuals with annual survival data available who were genotyped on a high-density SNP chip. **b** F_{ROH} for the full dataset including 7339 individuals with imputed genotypes. The orange dashed lines represent the medians. High-density genotyped individuals were chosen to be maximally unrelated and to maximise genetic diversity, which likely explains the missing right skew (i.e. a lack of inbred individuals) in their distribution (see Methods).

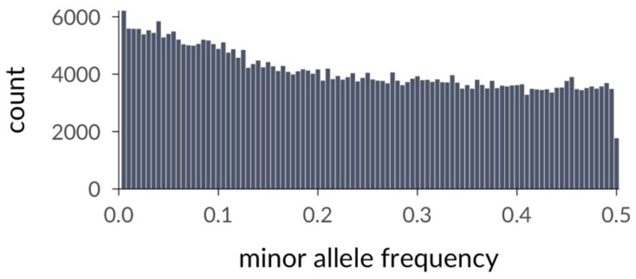

Supplementary Figure 2: Minor allele frequency distribution. Shown is the minor allele frequency (MAF) distribution across 417,373 imputed SNPs in 5,952 Soay sheep.

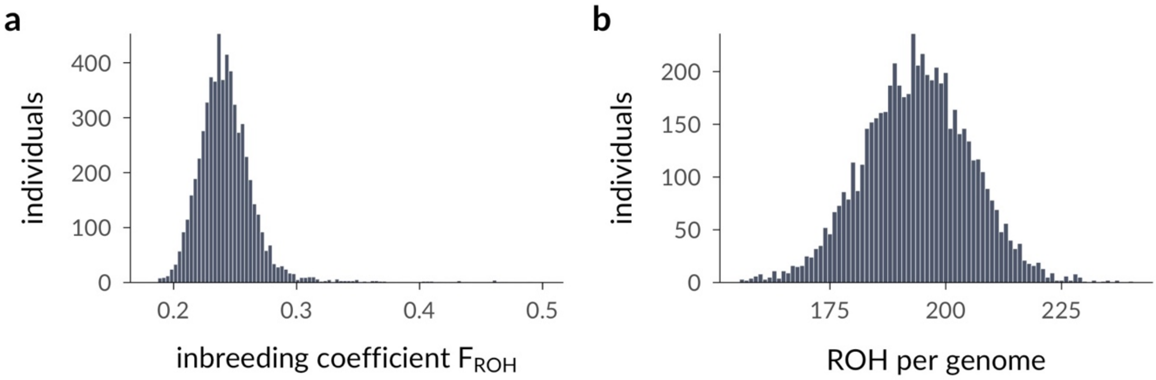

Supplementary Figure 3: Distribution of F_{ROH} and ROH. a Distribution of inbreeding coefficients F_{ROH} for 5952 individuals included in the survival analyses. **b** Distribution of the number of ROH per genome.

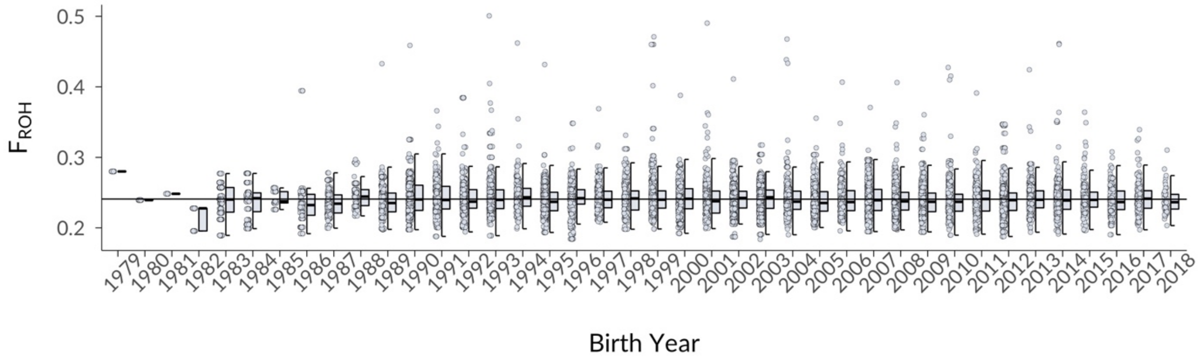

Supplementary Figure 4: F_{ROH} of individuals per birth year. The average inbreeding coefficient did not change over the course of the study period (β = 0, 95% CI [0,0], p = 0.588 for a linear model with F_{ROH} as response and birth year as numeric predictor). The plot shows the F_{ROH} of individuals as points, Tukey-boxplots per birth year to sum up the distribution of F_{ROH} in each year and the regression line of the linear model. Sample sizes n across 40 year (from 1979 to 2018) are 1979: 13, 1980: 15, 1981: 8, 1982: 23, 1983: 165, 1984: 164, 1985: 40, 1986: 137, 1987: 255, 1988: 145, 1989: 312, 1990: 656, 1991: 380, 1992: 532, 1993: 616, 1994: 279, 1995: 721, 1996: 464, 1997: 440, 1998: 299, 1999: 590, 2000: 626, 2001: 342, 2002: 608, 2003: 960, 2004: 359, 2005: 507, 2006: 463, 2007: 703, 2008: 703, 2009: 613, 2010: 435, 2011: 361, 2012: 501, 2013: 395, 2014: 337, 2015: 245, 2016: 230, 2017: 180, 2018: 54. The boxplots are standard Tukey boxplots (centre line = median, bounds of box = 25th and 75th percentiles, upper and lower whiskers = largest and smallest value but no further than 1.5 * inter-quartile range from the hinge).

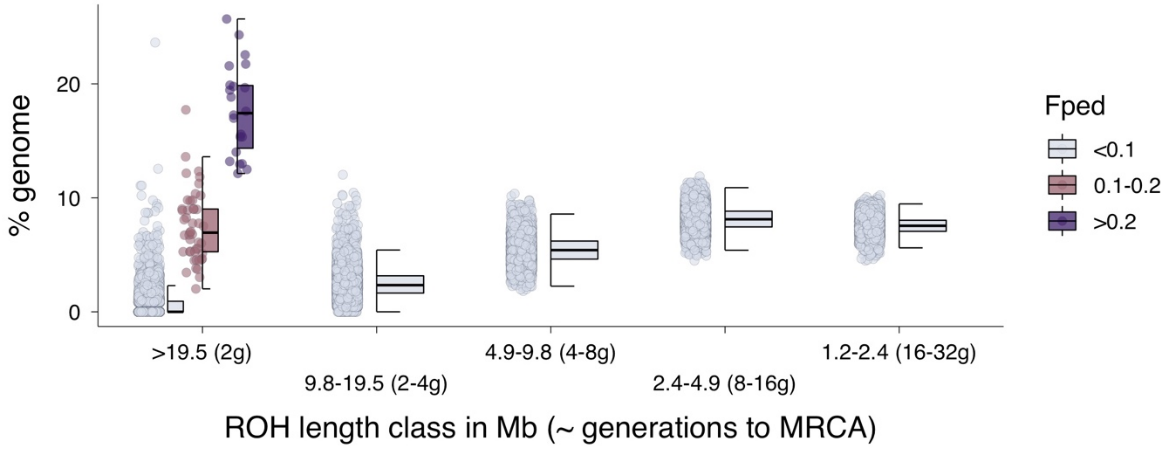

Supplementary Figure 5: Pedigree inbreeding among individuals with ROH > 19.5Mb. The figure shows the same data as Figure 1B with additional details on the pedigree inbreeding coefficient (F_{ped}) of individuals with long ROH > 19.5 Mb. F_{ped} was clustered into three classes comprising more outbred individuals (F_{ped} <= 0.1), moderately inbred individuals (0.1 < F_{ped} < 0.2) and strongly inbred individuals (F_{ped} >= 0.2). Most individuals with a large proportion of the genome in long ROH are inbred according to the pedigree.

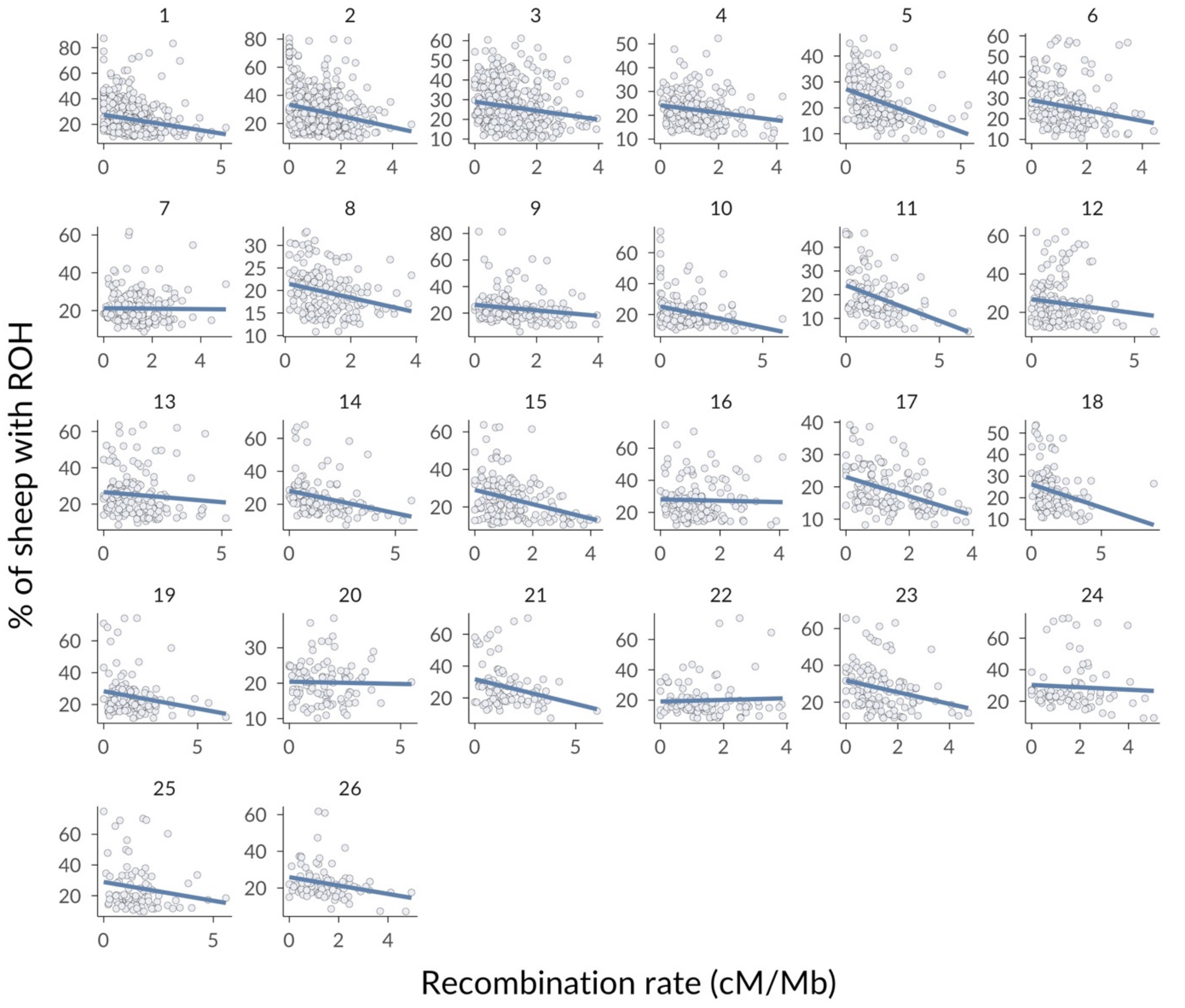

Supplementary Figure 6: ROH frequency and recombination rate in non-overlapping 500Kb windows across the genome. Shown is the mean frequency of ROH overlapping SNPs in 500Kb windows plotted against the rate of recombination in each window, facetted by chromosome, with linear model regression lines.

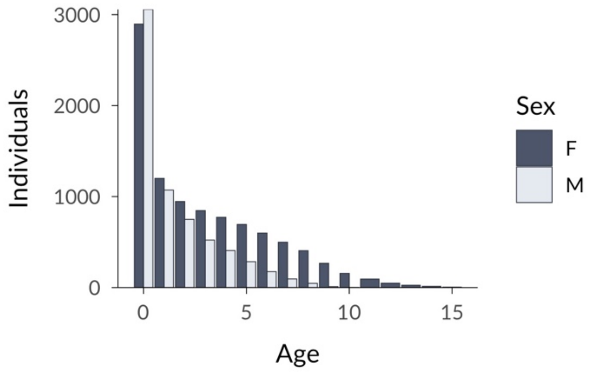

Supplementary Figure 7: Number of annual survival observations (individuals) per age class. Overall, the dataset consisted of 15889 observations from 5952 sheep.

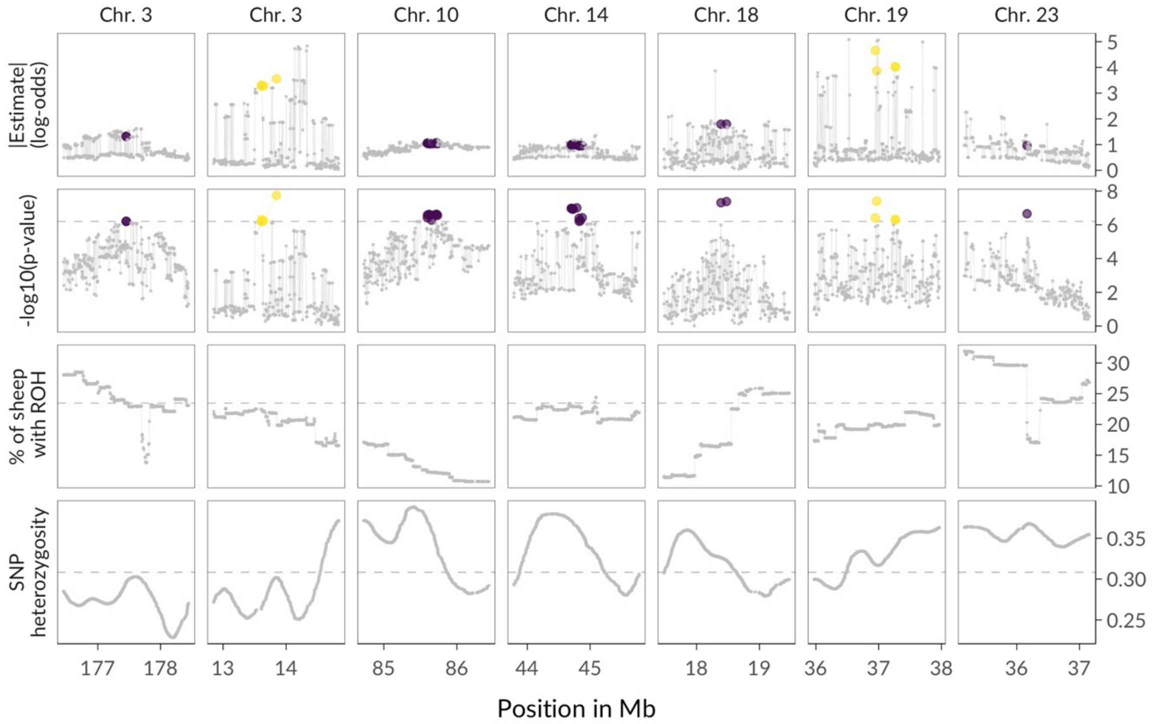

Supplementary Figure 8: GWAS estimates and genetic diversity around top GWAS hits. The first two rows show the GWAS model estimates (absolute) and p-values for all SNPs within a 2Mb window around the top SNP on each of the sevem genome-wide significant peaks located on six different chromosomes (see Figure 3). Purple points mark genome-wide significant negative ROH effects on survival, and yellow points mark genome-wide significant positive ROH effects on survival. Row three shows the proportion of individuals with ROH at each SNP position. Row four shows SNP heterozygosity which was smoothed using a Nadaraya-Watson kernel regression with a bandwidth of 100 SNPs. The dashed grey lines show the genome-wide significance level for p-values, and the genome-wide mean for the proportion of sheep with ROH and SNP heterozygosity.

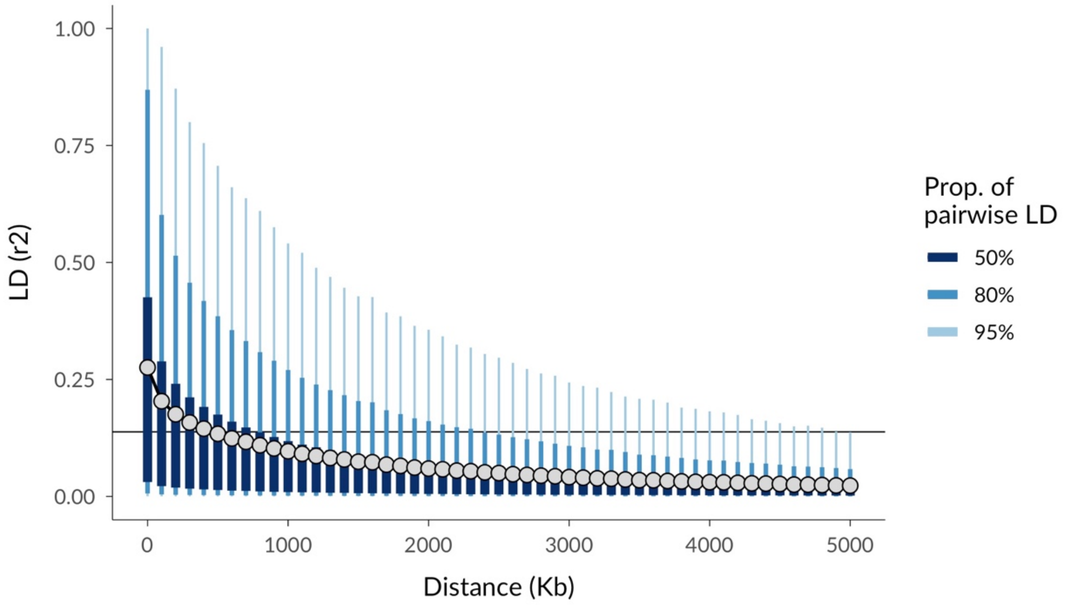

Supplementary Figure 9: LD decay in Soay sheep. LD was calculated based on (non-imputed) SNPs from the 50K SNP chip for all pairs of loci within each chromosome using –r2 in PLINK. The average of pairwise LD values was then calculated for loci pairs with increasing physical distance, in 100 Kb increments. The plot shows the distribution of LD r^2 values for loci up to 5 Mb apart. The proportion of pairwise LD comparisons per category is visualised using different shades of blue, with dark blue representing the interquartile range or middle 50% of data, and the lighter blue colors showing 80% and 95% of the data, respectively. The horizontal line is the indicator for the LD half decay. Consequently, LD half decays for loci approximately 600Kb apart (where the 6th mean LD point falls below the line).

Supplementary Tables

Chromosome	% SNPs correct	% SNPs imputed	N (Snps)
1	99.574	98.413	47318
2	99.547	99.180	41917
3	99.593	98.899	38265
4	99.454	99.440	20659
5	99.454	99.243	18545
6	99.315	99.584	19205
7	99.312	99.535	17647
8	99.726	99.707	15815
9	99.068	99.279	16517
10	99.342	99.541	15517
11	98.996	99.481	11567
12	99.420	99.708	13909
13	99.346	99.677	13626
14	98.979	99.425	10785
15	99.368	99.789	13897
16	99.528	99.655	11992
17	99.329	99.577	12598
18	98.904	99.467	11718
19	99.447	99.757	10230
20	99.109	99.609	9074
21	98.403	99.604	7879
22	99.007	99.706	9352
23	99.345	99.565	10004
24	98.628	99.660	6362
25	98.986	99.690	7530
26	99.152	99.614	7353

Supplementary Table 1: Cross-validation results for genotype imputation. To evaluate the accuracy of the genotype imputation, we masked genotypes from individuals which have been genotyped on the high-density SNP chip and used imputation to predict the masked genotypes. The table shows, for each chromosome, data summarised over the 10 cross-validation runs, in which genotypes for a single randomly chosen individual were masked and imputed per run. The second column shows the proportion of SNPs which were correctly imputed and column three shows the proportion of SNPs from the high-density array which could be imputed at all. The fourth column, N(SNPs), shows the number of SNPs on each chromosome.

Top 0.5% ROH deserts<br>ROH density measured in 500Kb running windows				
Chromosome	WinStart	WinEnd	% of individuals with ROH	N (SNPs)
11	58.5	59.0	4.45	103
9	0.0	0.5	5.02	13
21	49.0	49.5	5.39	26
11	58.0	58.5	5.50	110
11	59.0	59.5	5.78	85
9	0.5	1.0	5.96	93
11	61.5	62.0	6.79	105
11	57.5	58.0	6.95	94
26	37.5	38.0	7.23	95
21	48.5	49.0	7.23	48
26	37.0	37.5	7.40	114
11	57.0	57.5	7.41	116
14	5.5	6.0	7.45	110
11	59.5	60.0	7.53	101
11	60.0	60.5	7.72	148
11	56.5	57.0	8.06	89
11	61.0	61.5	8.09	108
22	9.5	10.0	8.10	101
5	5.0	5.5	8.18	96
11	60.5	61.0	8.20	118
17	71.5	72.0	8.21	75
17	64.0	64.5	8.38	98
22	12.0	12.5	8.38	115
22	11.5	12.0	8.39	89

Top 0.5% ROH islands<br>ROH density measured in 500Kb running windows				
Chromosome	WinStart	WinEnd	% of individuals with ROH	N (SNPs)
1	227.0	227.5	87.28	41
2	112.0	112.5	85.97	11
1	227.5	228.0	83.28	65
9	77.5	78.0	81.40	43
9	78.0	78.5	81.31	67
2	104.0	104.5	80.49	42
2	103.0	103.5	79.96	104
2	103.5	104.0	78.99	67
2	112.5	113.0	78.73	38
1	226.5	227.0	77.17	69
2	111.0	111.5	76.91	58
1	232.5	233.0	75.94	65
25	21.5	22.0	74.91	65
16	54.0	54.5	74.58	69
19	1.5	2.0	74.26	65
19	1.0	1.5	74.14	67
22	46.0	46.5	74.07	95
2	113.0	113.5	73.95	60
2	113.5	114.0	73.92	61
2	114.0	114.5	73.89	42
10	43.0	43.5	73.63	57
1	232.0	232.5	72.98	76
24	34.0	34.5	72.69	70
24	36.5	37.0	72.40	53

Supplementary Table 2: Top ROH deserts and islands. Shown are the top 0.5 % 500Kb regions with the lowest ROH density in our sample of 5952 sheep to the left and the top 0.5% 500Kb regions with the highest ROH density to the right. The start (WinStart) and end (WinEnd) of each window at the respective chromosome are given in Megabasepairs (Mb). The percentage of individuals with ROH was calculated as the average number of ROH overlapping the SNPs in a window. N (SNPs) shows how many SNPs were in a given window.

A

Term	Estimate	Std.Error	CI (2.5%)	CI (97.5%)	Standardization	Info	R2
Fixed effects							
Intercept	24.229	0.481	23.282	25.191			
Recombination rate (cM/Mb)	-2.307	0.121	-2.546	-2.063	(x-mean(x))/sd(x)	continuous	0.04, 95%CI [0.02, 0.07]
Heterozygosity	-6.97	0.121	-7.199	-6.744	(x-mean(x))/sd(x)	continuous	0.38, 95%CI [0.36, 0.40]
Random effects (variances)							
Chromosome	2.352	0.37	1.618	3.097		n = 26	
Residual	8.307	0.08	8.144	8.464			

B

Term	Estimate	Std.Error	CI (2.5%)	CI (97.5%)	Standardization	Info	R2
Fixed effects							
Intercept	31.677	0.532	30.545	32.892			
Recombination rate (cM/Mb)	-1.216	0.12	-1.479	-0.962	(x-mean(x))/sd(x)	continuous	0.01, 95%CI [0.00, 0.04]
Heterozygosity	-9.189	0.12	-9.439	-8.963	(x-mean(x))/sd(x)	continuous	0.53, 95%CI [0.50, 0.55]
Random effects (variances)							
Chromosome	2.623	0.38	1.834	3.353		n = 26	
Residual	8.269	0.083	8.11	8.443			

C

Term	Estimate	Std.Error	CI (2.5%)	CI (97.5%)	Standardization	Info	R2
Fixed effects							
Intercept	13.752	0.355	13.169	14.372			
Recombination rate (cM/Mb)	-2.353	0.101	-2.572	-2.165	(x-mean(x))/sd(x)	continuous	0.08, 95%CI [0.06, 0.11]
Heterozygosity	-3.395	0.101	-3.585	-3.202	(x-mean(x))/sd(x)	continuous	0.17, 95%CI [0.15, 0.19]
Random effects (variances)							
Chromosome	1.717	0.256	1.185	2.208		n = 26	
Residual	6.912	0.068	6.774	7.046			

Supplementary Table 3: Model estimates for a Gaussian mixed model of ROH prevalence in 500Kb windows across the genome. Table A shows the results for ROH > 1.2 Mb as used for the main analyses, table B shows the results for ROH > 0.3 Mb and table C shows the results for ROH > 3 Mb. Each table presents the model estimates of a mixed model with ROH prevalence as response, recombination rate and heterozygosity as fixed effects and chromosome as random effect. ROH prevalence has been estimated as the mean ROH per non-overlapping 500Kb window divided by the number of individuals. The last column reports the marginal R^2 of the model (row: Fixed effects) as well as the variance explained by recombination rate and heterozygosity (reported as semi-partial R^2, see Methods for details).

Term	Post.Mean	Std.Error	CI (2.5%)	CI (97.5%)	Standardisation	Info
Fixed effects						
Intercept	0.53 (1.7)	0.44 (1.55)	-0.32 (0.73)	1.42 (4.14)		
F_{ROH}	-0.91 (0.4)	0.14 (1.15)	-1.2 (0.3)	-0.63 (0.53)	(x * 10)-mean(x * 10)	continuous
LifeStage: EarlyLife	2.82 (16.78)	0.08 (1.08)	2.66 (14.3)	3 (20.09)		categorical (0=no, 1=yes)
LifeStage: MidLife	3.61 (36.97)	0.13 (1.14)	3.38 (29.37)	3.91 (49.9)		categorical (0=no, 1=yes)
LifeStage: LateLife	2.41 (11.13)	0.19 (1.21)	2.14 (8.5)	2.91 (18.36)		categorical (0=no, 1=yes)
Sex	-0.56 (0.57)	0.05 (1.05)	-0.66 (0.52)	-0.46 (0.63)		categorical (0=female, 1=male)
Twin	-0.74 (0.48)	0.07 (1.07)	-0.88 (0.41)	-0.61 (0.54)		categorical (0=no, 1=yes)
F_{ROH} * (LifeStage: EarlyLife)	-0.21 (0.81)	0.26 (1.3)	-0.72 (0.49)	0.3 (1.35)		
F_{ROH} * (LifeStage: MidLife)	0.12 (1.13)	0.38 (1.46)	-0.62 (0.54)	0.88 (2.41)		
F_{ROH} * (LifeStage: LateLife)	0.5 (1.65)	0.28 (1.32)	-0.05 (0.95)	1.05 (2.86)		
Random effects (variances)						
Birth year	0.79	0.18	0.59	1.24		n = 40
Capture year	2.57	0.17	2.36	2.97		n = 40
Individual	0.3	0.01	0.29	0.33		n = 5952
Add. genetic	0.3	0.02	0.28	0.34		Pedigree-based

Supplementary Table 4: Model estimates for the Bayesian binomial animal model of annual survival. Shown are the posterior mean, standard error, lower and upper credible interval on the logit (log-odds) scale with the exponentiated estimates (odds-ratios) in round brackets, and information about the standardisation of the variables. Life stage was fitted as a factor with four levels, lamb (age = 0, reference level), early life (age = 1,2), mid life (age = 3,4), late life (5+). The last column shows whether variables were fitted as continuous or categorical and how the levels for categorical variables were coded. For the random intercept effects of birth year, capture year and individual, the last column shows the number of groups. The last row in the table shows the estimates for the additive genetic variance, based on a pedigree-derived relationship matrix. The dataset underlying the model contained 15889 observations from 5952 individuals.

SNP	Chromosome	Position (Bp)	ROH status	Estimate (log-odds)	p-value	Allele1	Allele2
oar3_OAR3_177451112	3	177451112	AA	-1.32	6.25×10^{-7}	A	G
oar3_OAR3_13845652	3	13845652	GG	3.56	1.82×10^{-8}	A	G
oar3_OAR10_85720912	10	85720912	AA	-1.06	2.43×10^{-7}	A	G
oar3_OAR14_44787854	14	44787854	AA	-0.99	9.81×10^{-8}	A	G
oar3_OAR18_18475667	18	18475667	GG	-1.80	4.19×10^{-8}	G	A
oar3_OAR19_36967641	19	36967641	GG	3.87	3.90×10^{-8}	A	G
s23340.1	23	36164028	GG	-0.96	2.21×10^{-7}	G	A

Supplementary Table 5: SNPs with the strongest association between ROH status and annual survival at each GWAS peak. At each SNP location,

the effects of two binary fixed effects were tested, one for whether each allele being part of an ROH. The ROH status column shows the allelic ROH status, alongside the model estimate and the associated two-sided p-values from Wald Z-tests.
