# Supplementary material for: Genetic architecture and lifetime dynamics of inbreeding depression in a wild mammal
Source: Nat Commun. 2021 May 20;12:2972. doi: 10.1038/s41467-021-23222-9 (PMC8138023; doi:10.1038/s41467-021-23222-9)
Supplement: Supplementary file 3 — Reporting Summary [file 41467_2021_23222_MOESM3_ESM.pdf]

nature research

Corresponding author(s): Martin A Stoffel

Last updated by author(s): Mar 7, 2021

Reporting Summary

Nature Research wishes to improve the reproducibility of the work that we publish. This form provides structure for consistency and transparency in reporting. For further information on Nature Research policies, see our Editorial Policies and the Editorial Policy Checklist.

Statistics

For all statistical analyses, confirm that the following items are present in the figure legend, table legend, main text, or Methods section.

n/a	Confirmed	
☐	☒	The exact sample size (*n*) for each experimental group/condition, given as a discrete number and unit of measurement
☐	☒	A statement on whether measurements were taken from distinct samples or whether the same sample was measured repeatedly
☐	☒	The statistical test(s) used AND whether they are one- or two-sided<br>*Only common tests should be described solely by name; describe more complex techniques in the Methods section.*
☐	☒	A description of all covariates tested
☐	☒	A description of any assumptions or corrections, such as tests of normality and adjustment for multiple comparisons
☐	☒	A full description of the statistical parameters including central tendency (e.g. means) or other basic estimates (e.g. regression coefficient) AND variation (e.g. standard deviation) or associated estimates of uncertainty (e.g. confidence intervals)
☐	☒	For null hypothesis testing, the test statistic (e.g. *F*, *t*, *r*) with confidence intervals, effect sizes, degrees of freedom and *P* value noted<br>*Give P values as exact values whenever suitable.*
☐	☒	For Bayesian analysis, information on the choice of priors and Markov chain Monte Carlo settings
☐	☒	For hierarchical and complex designs, identification of the appropriate level for tests and full reporting of outcomes
☐	☒	Estimates of effect sizes (e.g. Cohen's *d*, Pearson's *r*), indicating how they were calculated

Our web collection on statistics for biologists contains articles on many of the points above.

Software and code

Policy information about availability of computer code

Data collection	No software was used for data collection
Data analysis	The complete data analysis pipeline can be accessed on GitHub: https://github.com/mastoffel/sheep_ID. The repository contains all code underlying the analyses and the versions of all packages contained in the renv.lock file.<br>Custom code was written with R 3.6.1, main packages used were: tidyverse 1.2.1, data.table 1.12.6, INLA 20.03.17, lme4 1.1-21, partR2 0.9.1, GenABEL 1.8.0, ggplot2 3.3.0.<br>Pedigree inference was done in Sequoia 2.0.7<br>Genotype imputation was conducted with AlphaImpute v1.98<br>Genetic data processing with PLINK v1.90b6.12

For manuscripts utilizing custom algorithms or software that are central to the research but not yet described in published literature, software must be made available to editors and reviewers. We strongly encourage code deposition in a community repository (e.g. GitHub). See the Nature Research guidelines for submitting code & software for further information.

Data

Policy information about availability of data

All manuscripts must include a data availability statement. This statement should provide the following information, where applicable:
- Accession codes, unique identifiers, or web links for publicly available datasets
- A list of figures that have associated raw data
- A description of any restrictions on data availability

We have deposited all data on Zenodo (http://doi.org/10.5281/zenodo.4609701).

Field-specific reporting

Please select the one below that is the best fit for your research. If you are not sure, read the appropriate sections before making your selection.

☐ Life sciences ☐ Behavioural & social sciences ☒ Ecological, evolutionary & environmental sciences

For a reference copy of the document with all sections, see nature.com/documents/nr-reporting-summary-flat.pdf

Ecological, evolutionary & environmental sciences study design

All studies must disclose on these points even when the disclosure is negative.

Study description	Genomic analysis of inbreeding depression in a wild population of Soay sheep.
Research sample	The analysed sample consisted of 15889 observations for 5952 Soay sheep (49% females, 51% males) born between 1979 and 2018 and sampled as part of a long-term study on the Hirta, the main island of the St. Kilda archipelago, Scotland. For all of these sheep, detailed genomic and survival data was available. The age of the individuals ranged from 0 to 16. Over 95% of new born individuals in the study area are tagged and sampled, and the sample is aimed at being representative for the overall Soay sheep population on St Kilda.
Sampling strategy	All individuals living in the Village Bay area of Hirta are monitored from birth to death via three expeditions per year.
Data collection	Lambs are tagged and sampled for genetic studies soon after birth. Some individuals that evade capture at lambing or immigrate into Village Bay are captured in a summer round up or by darting in the autumn. Survival is monitored by censusing the study population 30 times per year and searching for sheep that have died on each expedition. DNA is extracted from ear punches and samples genotyped on the Illumina 50K SNP ovine array. Data is collected by teams of researchers under the guidance of Jill Pilkington and Josephine Pemberton, with varying people recording measurements over the years.
Timing and spatial scale	The sample includes individuals born or sampled in Village Bay between 1979 and 2018. Sample collection since 1979 happened over three time periods a year to cover important periods in the life history cycle of sheep. From March to May, new lambs are born and tagged and sampled. In August, a large team of around 15 people catches the now few months old lambs alongside as many adult individuals as possible for phenotyping and to obtain DNA samples. The third sampling period from October to December happens during the sheep rut.
Data exclusions	Individuals of unknown birth or death year were excluded. Individuals for whom the genotyping failed were excluded.
Reproducibility	In a wild population the main measures ensuring reproducibility are large sample size of individuals studied over many years. The Soay sheep population is unmanaged, and we do therefore not conduct experiments.
Randomization	All individuals were included in a single analysis. Fixed effects of twin, age, sex and random effect of capture year, birth year, individual ID and genetic relatedness amongst others were included to control for covariates and variance components.
Blinding	Analysis was conducted using anonymous ID numbers which are different to the field tag codes. The data analyst (MAS) could therefore not connect the ID numbers in the dataset to observed sheep in the field.

Did the study involve field work? ☒ Yes ☐ No

Field work, collection and transport

Field conditions	The field site is in the NE Atlantic 60 km West of the Outer Hebrides. The long-term study was conducted over long time spans with varying weather conditions. In general, the climate on St. Kilda is oceanic with high rainfall and high humidity, with average temperature of 5.6 degrees celcius in January and 11,8 degrees celcius in July.
Location	Village Bay, Hirta. 57.8135° N, 8.5855° W. The elevation in the study area is below 200m at all points.
Access & import/export	The study site is within the UK so there are no restrictions to moving samples from Hirta to Edinburgh where they are analysed.
Disturbance	Disturbance to the sheep and other wildlife is short-lived and it is in the interests of the study to keep it so in this unmanipulated system.

Reporting for specific materials, systems and methods

We require information from authors about some types of materials, experimental systems and methods used in many studies. Here, indicate whether each material, system or method listed is relevant to your study. If you are not sure if a list item applies to your research, read the appropriate section before selecting a response.

Materials & experimental systems

n/a	Involved in the study
☒	☐ Antibodies
☒	☐ Eukaryotic cell lines
☒	☐ Palaeontology and archaeology
☐	☒ Animals and other organisms
☒	☐ Human research participants
☒	☐ Clinical data
☒	☐ Dual use research of concern

Methods

n/a	Involved in the study
☒	☐ ChIP-seq
☒	☐ Flow cytometry
☒	☐ MRI-based neuroimaging

Animals and other organisms

Policy information about studies involving animals; ARRIVE guidelines recommended for reporting animal research

Laboratory animals	No laboratory animals were used in the study.
Wild animals	Sheep were captured at birth or when older and tagged, weighed, measured and sampled for genetic and other analyses by taking ear punches and blood samples. As many animals as possible of both sexes were captured. Animals were checked for health and then released back to the wild as soon a possible after processing. The age of sheep ranged from 0 to 16 years.
Field-collected samples	Yes, all samples are collected in the field (see Location).
Ethics oversight	University of Edinburgh Animal Welfare Ethical Review Body and UK Home Office.

Note that full information on the approval of the study protocol must also be provided in the manuscript.
